# Prevalence of Noise-Induced Hearing Loss Among Tanzanian Iron and Steel Workers: A Cross-Sectional Study

**DOI:** 10.3390/ijerph16081367

**Published:** 2019-04-16

**Authors:** Israel P. Nyarubeli, Alexander M. Tungu, Bente E. Moen, Magne Bråtveit

**Affiliations:** 1 Centre for International Health, Department of Global Public and Primary Care, University of Bergen, Årstadveien 21, 5020 Bergen, Norway; Bente.Moen@uib.no; 2 Department of Environmental and Occupational Health, Muhimbili University of Health and Allied Sciences, P.O. Box 65015, Dar es Salaam, Tanzania; 3 Research Group for Occupational and Environmental Medicine, Department of Global Public and Primary Care, University of Bergen, 5020 Bergen, Norway; Magne.Bratveit@uib.no; 4 Department of Physiology, Muhimbili University of Health and Allied Sciences, P.O. Box 65001, Dar es Salaam, Tanzania; alextungu@yahoo.co.uk

**Keywords:** audiometry, occupational, noise-induced hearing loss, hearing threshold, exposed, iron and steel, Tanzania

## Abstract

Iron and steel factory workers in Tanzania are likely to develop noise-induced hearing loss (NIHL) due to exposure to high sound levels. Studies on hearing status in this population are lacking. The aims of this study were to determine prevalence of NIHL among iron and steel workers and compare hearing thresholds at different frequencies with a control group. We conducted a cross-sectional study among 221 iron and steel workers exposed to average noise level of 92 dB(A), compared with 107 primary school teachers recruited as controls and exposed to average noise level of 79.7 dB(A). We used a questionnaire-based interview to collect information on socio demographic characteristics and other confounding variables. Hearing loss was defined as hearing threshold levels ≥25 dB hearing loss in either ear at 3000, 4000 or 6000 Hz. The prevalence of hearing loss was significantly higher among the exposed group than among the controls, i.e. 48% and 31%, respectively. There were significant differences in hearing thresholds between the exposed and control groups at 3000, 4000, 6000, and 8000 Hz. Hearing loss was more frequent among workers exposed to higher noise levels than among the controls suggesting that iron and steel workers run a higher risk of developing hearing loss.

## 1. Introduction

Noise-induced hearing loss (NIHL) is an underestimated public health concern [1,2]. Globally, the magnitude of disabling hearing loss (above 40 dB) from all causes has increased in the past two decades from 120 to 466 million people from 1995 to 2018 [3,4]. Estimates of the prevalence of hearing loss related to noise exposure above 85 dB(A) vary in the range of 7–21% or higher [5]. Prevalence is estimated to be higher in the low and middle-income countries compared to the findings in other parts of the world [4]. This may be due to ongoing economic investments in industrialization coupled with challenges related to an inadequate public health policy, lack of regulatory frameworks and limited resources spent on preventive measures.

Studies highlight noise exposure as one major risk factor contributing to hearing loss [1,4,5]. Other suggested risk factors for hearing loss include increasing age, [6,7,8] smoking, [5] exposure to organic solvents, [9] the use of ototoxic medicines, [5,9] gender, vibration, genetics, ear surgery, ear infections and illnesses [5,10]. In addition, exposure to noise has been associated with increased risks of cardiovascular diseases and diabetes [5,11,12,13,14].

Studies conducted in Sub-Saharan Africa (SSA) have mainly focused on the mining sector and indicate, for instance, that the prevalence of hearing loss in this industry was 37% in Zimbabwe and 47% in Tanzania [15,16]. Despite the presence of hearing conservation programmes aimed at prevention, the prevalence of hearing loss was above 50% among gold miners in South Africa, while it was 21% among stone crushers in Ghana [8,17]. To our knowledge, there are no published studies on hearing loss in large iron and steel factories in SSA. One study among iron and steel mill workers in Western Africa, specifically Nigeria, found a hearing loss prevalence of 28% and 57% in the better and the worse ears, respectively [18]. This prevalence is almost twice as high as that found in the general adult population in Uganda [10]. One must take into account that the definition and presentation of hearing loss may differ from study to study [1]. Nevertheless, the prevalence of hearing loss is still alarming.

In Tanzania, like in other SSA countries, investments in the manufacturing industries, including iron and steel industries, create jobs for a significant large number of employees. Globally the demand for steel is increasing, and this sector has provided employment for 50 million people [19]. The construction of new infrastructures such as bridges, flyover exchange roads, buildings, towers and railways obviously create numerous workplaces. Although the construction of industrial-level infrastructure represents significant increase in economic assets across SSA, little is known about the prevalence of NIHL in these industries, and documentation is scarce to inform policy-makers and stakeholders working in preventive health services. In a recent study, the eight-hour average noise level among iron and steel workers in Tanzania was 92 dB(A), and 90% of the measurements were above the occupational exposure limit of 85 dB(A). The workers did not use hearing protection devices [20] implying that the workers are at increased risk of developing NIHL. There is a need for assessing NIHL in this working group with a view to developing a plan for implementation of a hearing conservation programme. Therefore, the aims of this study were to determine the prevalence of NIHL among iron and steel workers, and to compare the hearing thresholds at different frequencies between these workers and a control group exposed to a low level of occupational noise.

## 2. Materials and Methods 

### 2.1. Study Population

This cross-sectional study was conducted from June 2016 until June 2017 and involved permanent male workers from four iron and steel factories in Tanzania exposed to noise. Characteristics and details of noise-exposure assessments in these factories have been presented elsewhere [20]. The results showed a personal, mean equivalent noise exposure (*L*_EX,8h_) for these workers of 92.0 dB(A) [20]. 

Controls were male teachers from 34 public primary schools in Tanzania. This control group was chosen because they were expected to be exposed to low levels of occupational noise [5,21,22,23]. In the control group, 24 full-shift noise measurements from six primary schools were conducted using personal dosimeters (type 4448, Brüel and Kjær, DK-2850 Nærum, Denmark) attached to the teacher’s shoulder (ISO standard 9612:2009). The 8-hour equivalent noise exposure among these controls at work was 79.7 dB(A).

The sample size calculation was based on the estimated prevalence of hearing loss among workers exposed to loud noise at work. Since there was no available information about hearing loss among noise exposed workers or among the general population in Tanzania, the sample size was calculated based on a community baseline survey conducted in Uganda that found the prevalence of hearing loss among adults to be 12% [10]. In our study the effect of noise on hearing loss was hypothesized to be doubled i.e., 24%. To achieve 90% power and be able to detect a difference in hearing loss between noise exposed workers and a non-exposed group at a significance level of 0.05 (Using Open-Epi online calculator Version 3.3a, OpenEpi, Atlanta, GA, USA) [24], totally 230 exposed workers was needed. We added 10% to account for non-responders, providing a total sample size of 253 workers. 

### 2.2. Study Participants

**Figure 1:**
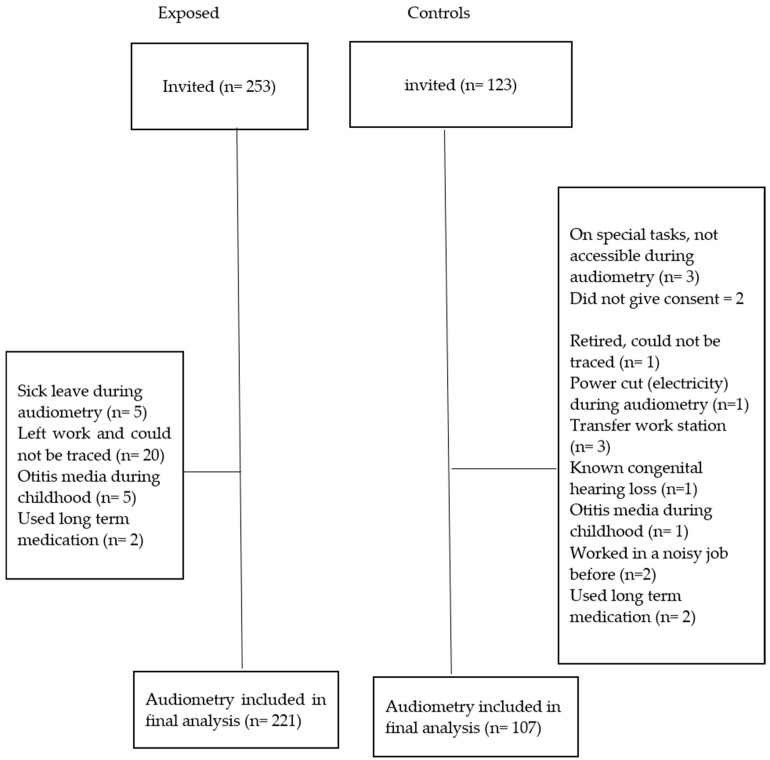
Flow chart describing the participants in the study of hearing loss among exposed iron and steel factory workers (*n* = 221) and controls—primary school teachers (*n* = 107) in Tanzania.

A total of 376 permanent workers (253 from four iron and steel factories and 123 teachers from 34 public primary schools) were randomly selected by using a table of random numbers from the provided list of workers and were invited to participate in the study (Figure 1). Workers list was provided by the respective employers. We held meetings with both the management for each factory and the administration at the public primary school where we presented the purpose of the project and asked for a research permit. Each of these partners referred us to a contact person who helped the research team in the planning of the research activities. The study participants were informed of the purpose of the project and those who agreed to participate, provided written consent. This paper presents the audiometry results.

We used information collected through a structured interview with both exposed participants and controls to exclude the following categories of workers from the data analysis: those with congenital hearing loss, history of otitis media during childhood and those reported to have worked in noisy job among the controls. In addition, we excluded those who reported using long-term medication because the participants did not have adequate knowledge of the type of medication they used. Thus, we ended with a total of 328 (221 exposed and 107 controls) persons taking audiometric measurements (Figure 1).

The participants were instructed to avoid areas with high level of noise for a minimum of 12 h prior to audiometric examinations to minimize the possibility of temporary threshold shift (TTS). The duration since last occupational noise exposure (free noise exposure) was recorded before the audiometric test was administered [25,26].

### 2.3. Interview Questionnaire and Checklist

A structured interview questionnaire was used to collect information on demographic characteristics and factors that may influence hearing loss. These included age (in years), number of years of employment, history of noise exposure at work (yes/no), current smoking (yes/no), present use of long-term medication (yes/no), exposure to chemicals/organic solvents (yes/no), use of hearing protection while working in noisy areas (yes/no), ear infections as a child or adult (yes/no), head injury/trauma (yes/no), tinnitus (yes/no). In addition, information about otitis in childhood (yes/no), known congenital hearing loss (yes/no), any relatives with hearing loss and any history of ear-related medical condition (diabetes and hypertension) was collected. This information was collected before the audiometry day and used to exclude participants before audiometry (Figure 1).

Prior to otoscopy, but on the same day as the audiometry, participants were interviewed using a checklist indicating whether they had symptoms of upper respiratory infections (e.g., running nose) (yes/no), ear discharge (yes/no), time and date they left work (hours) and the most recent time they were exposed to high noise at a level that made it difficult to communicate. Afterwards, otoscopy was performed by an occupational physician; in circumstances when the ear canal was completely obstructed with wax or cerumen, the latter were removed, and a new appointment was scheduled for audiometry. This also applied to participants with upper respiratory infections; the test was postponed until they were asymptomatic.

### 2.4. Pure Tone Audiometry

Audiometric measurements (pure tone audiometry) were conducted in an ear-screening locally-constructed booth in a quiet room at the headquarters of the Occupational Safety and Health Authority (OSHA) in Tanzania. The same technical personnel conducted all audiometric tests using a standardized protocol. Background noise in the test booth was monitored by a calibrated hand-held Sound Level Meter (Brüel and Kjær, type 2250), and checked for conformity with ISO 8253-1:2010 standard [27]. The highest background noise level (L_max_) in the booth was 51 dB at 31.5 Hz. For best results, audiometry was conducted in the morning before any work exposure. In addition, the city is less noisy in the morning compared to other times of the day when the participants could potentially be exposed to a higher level of environmental noise. Pure tone audiometry was conducted using an Interacoustics AD226 (Interacoustics, DK-5500, Middelfart, Denmark) with Amplivox Audiocup earphones having lower test limit of −10 dB. The equipment was pre-calibrated. Test frequencies were 250–8000 Hz in the order starting with 1000, 2000, 3000, 4000, 6000, 8000, 500, 250 and finish at 1000 Hz [27]. A manual test procedure was used in compliance with ISO 8253-1:2010 [26,27,28].

### 2.5. Data Analysis

Descriptive statistics were presented as mean and standard deviation or percentage. Chi-square and independent samples *t*-tests were used to compare categorical and continuous descriptive variables, respectively. NIHL was defined as hearing threshold level ≥25 dB hearing loss in either ear at 3000, 4000 or 6000 Hz [29].

Potential determinants of hearing loss were identified. Age was categorized into three age groups (tertiles) based on the age distribution among the controls. Duration of work was categorized arbitrarily into three groups (≤2 years, 2–10 years, 11–37 years). History of ear-related medical conditions (diabetes, hypertension, ear infections and head injury) was combined into a dichotomized variable (yes/no), current smoking (yes/no), relatives with hearing impairment (yes/no), tinnitus (yes/no) and previous noisy work (yes/no). A chi-square test was used to explore the relationship between these variables and hearing loss in exposed participants compared with controls. 

The intercorrelation between participant’s age and duration of work determinants was tested with the Pearson correlation test. In multiple regression analyses, we chose the determinant that contributed most to the hearing loss. 

We used log binomial regression models with a 95% confidence interval (CI) to ascertain differences in hearing loss (yes/no) between exposed and controls within each age strata and within the total group of workers while adjusting for the significant determinants selected from Chi-square analyses and the correlation test.

We computed the mean hearing threshold for the different test frequencies for both exposed participants and controls, as well as for the three age groups within the main exposure groups. For each test frequency, multiple linear regression was used to analyze for differences between exposed and controls, while adjusting for age as a continuous variable, previous noisy work and history of ear-related medical condition.

The exposed group had a mean exposure duration of 5 years (range: 0–24 years) and a L_Aeq8h_ of 92 dB(A) [20]. Within the three age groups (≤2 years, 2–10 years, 11–37 years) the mean duration of exposure in these factories were 1, 5, and 17 years, respectively. We calculated the predicted noise-induced permanent threshold shift (NIPTS) corresponding to these three mean exposure durations according to ISO 1999 section 6.3, that provides a formula and method that predicts NIPTS at 1000, 2000, 3000, 4000 and 6000 Hz as a function of the logarithm of exposure duration (*d*) (in years), and the square of noise exposure level (L_Aeq8h_), with frequency specific constants *u*, *v*, and L_0_ (a sound pressure level, defined as a function of a given constant value for each frequency in decibels [30]:

NIPTS = [*u* + *v*log_10_ (*d*)] (L_Aeq8h_ − L_0_)^2^ (1)

We used IBM SPSS Statistics, Version 25 (Allen & Unwin, 83 Alexander Street, Crown Nest, NSW, Australia) for data analysis and set a parameter of *p* < 0.05 as statistical significance. NIPTS was estimated using Microsoft Excel (Office 365, Microsoft Corporation, Redmond, WA, USA).

### 2.6. Ethical Clearance

We obtained ethical clearance from The Regional Committee of Medical and Health Research Ethics (REK-VEST) in Norway (number 2016/635/REK sør-øst dated 20 May 2016); and later from The Muhimbili University of Health and Allied Sciences (MUHAS) Ethics Committee in Tanzania number 2016-06-24/AEC/Vol. XI/38 dated 24 June 2016. Each iron and steel factory and primary school administration was contacted individually, and all of them granted permission to conduct the study. Individual participants were contacted and informed about the research objectives and activities to be conducted and gave written consent. Information that was collected was treated as confidential and was not accessed by unauthorized parties. We used participants’ identification instead of names in data collection, processing and analysis.

## 3. Results

The participation rate was 87% for both the exposed and controls. The exposed group was significantly younger than the controls (independent sample *t*-test; *p* < 0.001) (Table 1). There was a significant difference between exposed and controls for the three descriptive variables; age group, duration of work and previous noisy work, (Chi square test; *p* < 0.001) but not for the other variables i.e., current smoking, tinnitus, relative with hearing impairment and history of ear-related medical condition (Table 1). Among the exposed, 67% of the workers fell in the youngest age group (18–35 years) (Table 1). In addition, there were significant differences in hearing loss between exposed and controls for the four determinants—age group, duration of work, previous noisy work and history of ear-related medical condition (Chi-square test; *p* < 0.05). The overall prevalence of hearing loss was significantly higher (Chi square test, *p* = 0.003) among exposed workers (48%) than among the controls (31%) (Table 2). 

Hearing loss increased with advancing age among both exposed and controls (Table 2). Within the age-groups, there were significant differences in hearing loss between exposed and the controls for the youngest and middle-aged group (Chi square test, *p* 1 = 0.002; *p* 2 = 0.013) but not for the older age group (Table 2). 

Results from the log binomial regression model, adjusted for age, previous noisy work and history of ear-related medical condition, showed a statistically higher risk of hearing loss among exposed workers compared to controls, with a prevalence ratio of 1.3. When performing the analysis within each age stratum, the youngest age group (18–35 years) had the highest prevalence ratio (2.5), although it was not statistically significant (Table 2).

**Figure 2:**
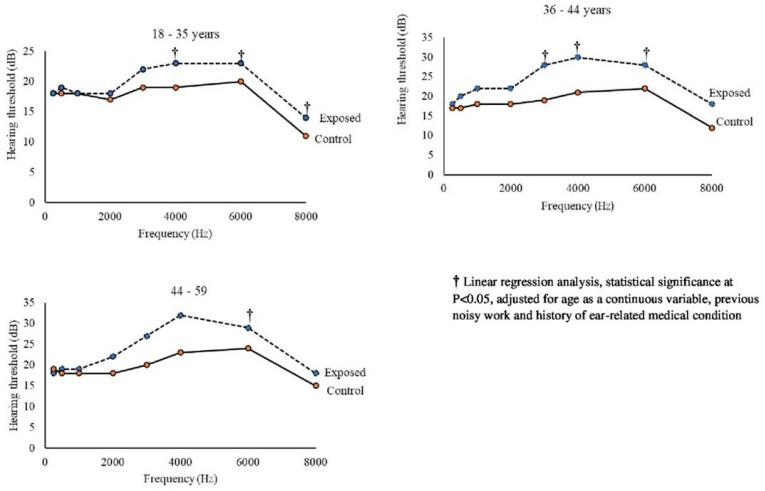
Hearing threshold of noise-exposed male workers (*n* = 221) (dotted lines) compared with male controls (*n* = 107) (solid lines) in Tanzania, stratified into age groups (triplets).

The mean hearing threshold between exposed and control workers at 3000, 4000 and 6000 Hz differed significantly (independent samples *t*-test, *p* < 0.05) (Table 3). In linear regression analyses within each age stratum, there were significant differences in hearing threshold between exposed and controls for the frequencies 4000 and 6000 Hz within the youngest age group (18–35 years) adjusting for age as a continuous variable, previous noisy work and history of ear-related medical condition (Figure 2). In analogous analyses, the hearing threshold for the frequencies 3000, 4000 and 6000 Hz were significantly different in the 36–43 years age group, and in the age group 44–59 years, only the frequency 6000 Hz was significantly different (Figure 2).

Table 4 shows the age-stratified differences in hearing thresholds for the different test frequencies in exposed and controls. The regression coefficients show that for the frequencies with significant findings, the difference between exposed and controls was about 3–6 dB among the youngest age group, 4–6 dB in the middle-aged group and about 10 dB for the oldest age group.

**Figure 3:**
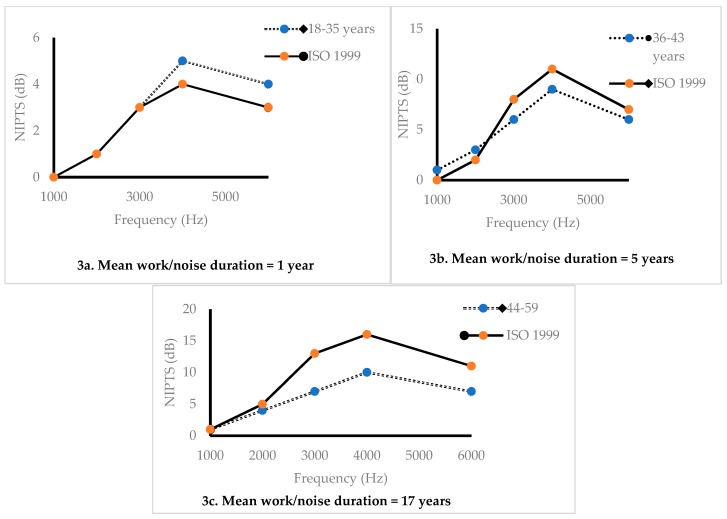
(**a**–**c**) Mean of the measured hearing threshold by audiometry (dotted lines) and median noise-induced permanent threshold shift (NIPTS) predicted by ISO 1999 (solid lines) for the three age-groups of iron and steel workers in Tanzania exposed to an average noise level of L_Aeq8h_ of 92 dB(A) for the mean duration of noise exposure within each age group.

The mean hearing threshold among the participants in the 18–35 age group was similar to the predicted NIPTS according to ISO 1999 at the lower frequencies (1000, 2000 and 3000 Hz), while it was about 1 dB higher than ISO 1999 for the higher frequencies (4000 and 6000 Hz) (Figure 3a). For the 36–44 and 45–59 age groups, the hearing threshold for the higher frequencies were lower (3, 1 dB and 6, 4dB lower, respectively) for same frequencies than that the NIPTS predicted by ISO 1999 (Figure 3b,c). 

## 4. Discussion

We found a higher prevalence of hearing loss among Tanzanian iron and steel factory workers compared to controls i.e., 48% vs. 31% respectively. In addition, a comparison of hearing thresholds between the two groups for the frequencies 4000 and 6000Hz revealed significant differences. To our knowledge, this is the first study in SSA to document the prevalence of hearing loss among workers exposed to noise in iron and steel factories.

In the present study, we found a significantly higher prevalence of NIHL among iron and steel workers than the controls. The noise exposed workers were exposed to a mean noise level of 92 dB(A), without using hearing protection devices [20]. At this noise level, it is likely that the workers develop NIHL [31,32]. A study conducted among Indian iron and steel workers exposed to noise levels above 90 dB(A) found an even higher prevalence of NIHL than we found. Over 90% of the workers engaged in casting and forging had hearing loss in the higher frequencies i.e., 4000 and 6000 Hz [33]. This is likely due to differences in the nature of work, including tasks and tools used during the steel production process. For example, the Indian study was done in small and medium factories with the forging and casting tasks frequently characterized by impulse noise that might cause hearing damage at higher frequencies [34]. By contrast, our study was done in larger-scale factories with a relatively higher level of mechanization. Another study done in Nigeria also found higher prevalence in the worse ear (57%) among steel mill workers exposed to 75–93 dB(A), with pure tone averages of 30 dB, 31 dB and 32 dB for the finishing, mill floor and mechanical departments respectively, as compared to a pure tone average of 21 dB among administrative workers with lower noise exposure (49 dB(A)) [18]. In Nepal, the prevalence among workers in a steel factory was comparable to our study, i.e., 40% and 46% for the right and left ear, respectively. However, the study excluded workers over 45 years and information on factory characteristics were not available [35]. Another study done in Nepal among 115 small-scale metal industry workers and 123 controls found lower prevalence for the exposed (30%) and only 4% for the controls [36]. The difference in prevalence between the Nepal study and our study may be due to the definition used to define hearing loss [1,36]. However, the high prevalence presented based on these studies suggests that noise exposure among iron and steel workers contribute substantially to hearing loss [37]. 

Age is one of the main factors for the development of hearing loss. To adjust for age can be difficult in statistical analyses. In the present study, we stratified the working population into three age groups and found a borderline increased risk for hearing loss among the younger age group (18–35 years), and significant differences between exposed and controls in hearing thresholds for the frequencies of 4000 and 6000Hz. The significant difference in the dip for the 4000 and 6000 Hz frequencies is a sign indicating hearing loss due to noise exposure in this age stratum [17,18,38,39]. Similar findings have been shown among gold miners in South Africa where the greatest difference in hearing threshold between age strata was found among the younger age group (16–40 years) at the noise dip of 4000 Hz [7]. Therefore, it is essential that noise control measures, including hearing conservation programmes should be established particularly to protect workers from developing NIHL.

In this study we found higher estimates of NIPTS than predicted by ISO 1999 standard for 18–35 years at frequencies of 4000 and 6000 Hz. These frequencies are likely to be affected by noise exposure [40]. The characteristics of noise, size of ear canal and other factors determines the location of notch for the higher frequencies [41]. However, the notch at these frequencies and especially at 4000 Hz is an established clinical sign and may be valuable in confirming the diagnosis of NIHL [40,42]. In addition, Our NIPTS estimates, though generally lower than that of ISO 1999 predictions, show similar patterns especially at higher frequencies. This result differs from a study conducted in United States which reported estimates in agreements to that of ISO 1999 [43]. The lower results and estimates from our study may be explained partly by differences in reference population characteristics. ISO 1999 standard was prepared based on populations from developed and industrialized countries such as United States and with steady state noise [30], which it is difficult to compare results to our study that had mixed noise characteristics. However, although the hearing threshold in the age range 44–59 years was somewhat lower than predicted from the ISO 1999, the overall results suggests that noise exposure among the iron and steel workers leads to an increased risk of NIPTS. 

The control group in our study had a significant lower prevalence of hearing loss compared to the exposed workers at higher frequencies. The measured hearing loss in this group was lower than that recorded among the controls in South African miners study for the higher test frequencies i.e., 31% versus 46% respectively [8]. The control group in the South Africa study was the administration group, and this makes it difficult to compare with our study. Moreover, the control group in the South Africa study was not screened for previous noise exposure as we did in our study. The participants in our control group were screened for several factors responsible for hearing loss and were thus expected to have low prevalence. This indicates that there might be factors other than noise that may have contributed to the hearing loss in the South African study. In Tanzania, there are no published data on community hearing profile among adults. Community studies conducted in other African countries such as Nigeria and Egypt found a lower prevalence of hearing loss (defined as hearing threshold >25 dB) than we found i.e., 18% and 16%, respectively [44]. However, in these community studies, there is no information on noise exposure profile among the participants, and this makes it difficult to compare with the control group in our study. Based on the selection of examined workers, including the control group, we think that it is likely that occupational noise exposure has contributed to the difference in hearing loss between our two groups.

Strengths of this study are the high response rate among the participants, the use of a control group from workplaces with low sound levels, and the use of standardized methods for audiometry. In addition, it was possible to control for the effect of age in hearing thresholds by stratification of age groups while adjusting for age as a continuous variable within the age strata. The statistical analyses made it possible to adjust for potential confounding factors related to hearing loss, such as current smoking, previous noise exposure, tinnitus, history of ear-related medical conditions, duration of work and relatives with hearing impairments. The use of calibrated research equipment and devices together with adherence to the novel procedure related to audiometry testing and ISO 8253-1:2010 standard for ambient noise improved the findings. 

Our study had some limitations; The design of the study was cross-sectional, and this reduces the possibility to conclude regarding the causal relationship between noise at work and hearing loss. Still, this study indicates that the sound levels are of importance to the registered hearing losses in this working population, as the frequencies involved are in the upper frequency area and the sound levels measured were above 85 dB(A). A longitudinal study would have provided a better exposure-effect association. Information collected through interview questionnaire might introduced recall bias. To minimize this bias, we used the same trained research personnel and method for both the exposed and the controls. In addition, in many societies today, people listen to music at high volume levels, and this may affect their hearing ability. We have limited information about leisure time exposure to noise among our study participants, but we have no reason to believe that the workers in iron and steel factories are more exposed to leisure time sound than are the control workers. In addition, iron and steel workers spent most of their time during the day at work. Lastly, it was impractical to monitor workers at their homes before audiometry.

## 5. Conclusions

Based on these findings, this study should be a wake-up call for stakeholders in the establishment and should serve to encourage the implementation of noise control measures such as the use of hearing protection devices in these workplaces. The information we found on the high prevalence of hearing loss may be used by policy and decision-makers in awareness creation programmes aimed at noise control such as establishing hearing conservation programmes and preventive services among working populations exposed to noise [33]. 

## Figures and Tables

**Table 1:** Descriptive characteristics of the participants in the study among noise-exposed (*n* = 221) and control (*n* = 107) workers in Tanzania.

Characteristics	Descriptive		*p*-Value
	Exposed (*n* (%))	Controls (*n* (%))	
Age: Mean (SD)	32 (8)	40 (7)	<0.001 ^a^
Age group (years) (group mean for Exposed))			
18–35 (27)	149 (67.4)	36 (33.6)	<0.001 ^b^
36–43 (39)	58 (26.2)	37 (34.6)	
44–59 (47)	14 (6.3)	34 (31.8)	
Total	221 (100.0)	107 (100.0)	
Duration of work (years) (group mean for Exposed)			
≤2 (1)	86 (38.9)	-	<0.001 ^b^
3–10 (5)	108 (48.9)	27 (25.2)	
11–37 (17)	27 (12.2)	80 (74.8)	
Current smoking			
no	183 (82.8)	96 (89.7)	
yes	38 (17.2)	11 (10.3)	0.07
Previous noisy work			
no	178 (80.5)	107 (100.0)	
yes	43 (19.5)	-	<0.001 ^b^
Tinnitus			
no	202 (91.4)	104 (97.2)	
yes	19 (8.6)	3 (2.8)	0.06
Relative with hearing impairment			
no	199 (90.0)	96 (89.7)	
yes	22 (10.0)	11 (10.3)	0.9
History of ear-related medical condition			
no	176 (79.6)	93 (86.9)	
yes	45 (20.4)	14 (13.1)	0.01 ^b^

^a^ independent samples *t*-test; ^b^ Chi-square test.

**Table 2:** Prevalence of hearing loss among exposed (*n* = 221) and control (*n* = 107) workers in Tanzania.

Variable	Hearing Loss ^a^ (*n* (%))			
	Exposed	Controls	Chi-Square Test (*p*-Value)	Prevalence Ratio 95% Confidence Interval) ^†^
Age group (years)				
18–35	63 (42.3)	5 (13.9)	0.002 *	2.5 (0.93, 6.76)
36–43	34 (58.6)	12 (32.4)	0.013 *	1.7 (0.79, 3.47)
44–59	10 (71.4)	16 (47.0)	0.124	1.5 (0.58, 3.70)
All	107 (48.4)	33 (30.8)		1.3 (1.10, 1.62)

^a^ Hearing loss defined as ≥25 dB in either ear at 3000, 4000 or 6000 Hz; ^†^ log-binomial analysis within each age group, adjusted for age as a continuous variable, previous noisy work and history of ear-related medical condition; * *p* < 0.05.

**Table 3:** Hearing threshold of the worse ear among noise exposed and controls for the tested frequencies.

Workers’ Group	Number of Workers	Mean Hearing Thresholds in Decibel (dB) for Each Frequency							
		250	500	1000	2000	3000	4000	6000	8000
Exposed	221	17.0 (6.1)	19.0 (5.2)	19.4 (6.0)	19.6 (7.3)	23.6 (8.9) ^a^	25.0 (9.7) ^a^	24.3 (10.6) ^a^	16.0 (9.7) ^a^
Control	107	17.2 (5.5)	18.0 (5.7)	19.7 (5.2)	18.8 (8.1)	20.8 (8.0)	21.7 (8.8)	19.6 (9.2)	13.6 (9.8)

^a^ independent samples *t*-test, *p* < 0.05.

**Table 4:** Hearing threshold at the tested frequencies stratified by age groups among exposed iron and steel factory workers (*n* = 221) and controls (*n* = 107) in Tanzania.

Age Group	Audiometry Frequency (Hz)													
	250		500		1000		3000		4000		6000		8000	
	*β*	95% CI	*β*	95% CI	*β*	95% CI	*β*	95% CI	*β*	95% CI	*β*	95% CI	*β*	95% CI
18–35														
	0.16	−2.31, 2.63	−1.54	−3.47, 0.39	−0.53	−2.57, 1.52	−3.05	−6.19, 0.11	−4.94 *	−8.57, −1.31	−5.84 *	−10.16, −1.52	−4.90 *	−8.66, −1.14
36–43														
	−0.14	−3.06, 2.77	−1.92	−4.63, 0.79	−1.45	−4.54, 1.64	−5.70 *	−10.11, −2.27	−6.37 *	−10.93, −1.81	−4.32 *	−8.73, 0.09	−3.36	−7.51, 0.79
44–59														
	−0.72	−5.51, 4.07	−2.47	−6.52, 1.59	−1.07	0.67, −6.20	−6.85	−14.66, 0.95	−4.95	−13.23, 3.33	−10.22 *	−18.87, −1.58	−6.00	−15.41, 3.41

Linear regression analysis, adjusted for age as a continuous variable, previous noisy work and history of ear-related medical condition. * Statistical significant at *p* < 0.05.
